# Childbirth care by health professionals: conflicting practices in obstetrics

**DOI:** 10.1590/0034-7167-2023-0129

**Published:** 2024-12-16

**Authors:** Greici Naiara Mattei, Gabriela Marcellino de Melo Lanzoni, Giovana Dorneles Callegaro Higashi, Taís Regina Schapko, Maria Aparecida Baggio

**Affiliations:** I Universidade Estadual do Oeste do Paraná. Cascavel, Paraná, Brazil; II Universidade Federal de Santa Catarina. Florianópolis, Santa Catarina, Brazil; III Universidade Federal de Santa Maria. Palmeira das Missões, Rio Grande do Sul, Brazil; IV Universidade Estadual do Oeste do Paraná. Foz do Iguaçu, Paraná, Brazil

**Keywords:** Midwifery, Nursing, Pregnant Women, Health Personnel, Obstetrics., Partería, Enfermería, Mujeres Embarazadas, Personal de Salud, Obstetricia.

## Abstract

**Objectives:**

to understand the perceptions of women and health professionals regarding childbirth care at a teaching hospital in the western state of Paraná, Brazil.

**Methods:**

this qualitative study employed Grounded Theory, conducted in an obstetric care service with 38 participants (women and health professionals) through semi-structured interviews.

**Results:**

limitations in physical infrastructure and management of care, along with the women’s limited knowledge about the childbirth process and the decision-making and guidance of professionals, show conflicting obstetric practices-a discrepancy between good practices and obstetric violence. Training in obstetric nursing and active participation in care, alongside the presence of a companion, were identified as intervening conditions and strategies in the process.

**Final Considerations:**

childbirth care is characterized by dichotomous practices. While some professionals base their practices on scientific evidence, others rely on teachings and experiences from the time of their training.

## RESUMEN

**Objetivos:**

comprender la percepción de mujeres y profesionales de salud sobre la partería en un hospital-escuela del oeste del estado de Paraná.

**Métodos:**

investigación cualitativa del tipo Teoría Fundamentada en Datos, realizada en servicio de atención obstétrica con 38 participantes (entre mujeres y profesionales de salud) mediante entrevistas semiestructuradas.

**Resultados:**

las limitaciones de la estructura física y gestión de la atención, el incipiente conocimiento de mujeres sobre parto y el direccionamiento y toma de decisión de profesionales indican la conformación de prácticas obstétricas opuestas, una divergencia entre buenas prácticas y violencia obstétrica. La formación en enfermería obstétrica y participación activa en el cuidado relacionada a la presencia del acompañante fueron condiciones intervinientes y estrategias en el proceso.

**Consideraciones Finales:**

la partería es conformada por actuaciones dicotómicas. Mientras algunos profesionales sustentan sus prácticas basadas en evidencias científicas, otros las pautan en enseñanzas y experiencias relativas a la época de su formación.

## INTRODUCTION

Childbirth, a natural and physiological event, has been influenced by the biomedical model and has become susceptible to various interventionist practices^(1)^. To ensure a positive childbirth experience, the World Health Organization (WHO) has developed the Intrapartum Care Guide, which includes a series of recommendations for obstetric care professionals and women in labor^(2)^.

Appropriate practices position the woman as the central figure in care, recognizing and considering her needs without being affected by the impositions of health professionals or the institution where the patient is located^(3)^. Woman-centered care during childbirth allows her to decide on obstetric care in accordance with current recommendations, leading to satisfaction with the care received and a positive childbirth experience. These conditions influence her decision to seek the same obstetric health service again or recommend it to other pregnant women^(4)^.

The environment of the childbirth setting, the guarantee of privacy, respect for choices, provision of information to women to enable informed consent, and the actions of health professionals involved in the parturition process, among other factors, affect the childbirth experience^(5)^. Additionally, the cooperation of the multiprofessional team and the participation of professionals who are up-to-date and open to innovation have contributed to a new care model oriented toward appropriate practices^(6)^.

It is important to note that in Brazil, although the implementation of WHO recommendations is identified in some settings, an obstetric care model not based on scientific evidence persists in others, reflecting disrespectful care. In other words, there is a coexistence of recommended and non-recommended practices without scientific basis, such as the Kristeller maneuver, routine episiotomy, and restriction of movement or food intake^(7,8)^. Given this, the question arises: How do women and nursing and medical professionals understand childbirth care in a teaching hospital in the western state of Paraná?

## OBJECTIVES

To understand the perceptions of women and health professionals regarding childbirth care at a teaching hospital in the western state of Paraná, Brazil.

## METHODS

### Ethical Aspects

This study is derived from a larger project titled “*Rede Mãe Paranaense na perspectiva da usuária: o cuidado da mulher no pré-natal, parto, puerpério e da criança*” (Paranaense Mother Network from the User’s Perspective: Women’s Care in Prenatal, Childbirth, Postpartum, and Childcare), approved by the Research Ethics Committee involving Human Subjects of a public higher education institution in northern Paraná. It was conducted in accordance with Resolution No. 466/2012 of the National Health Council and Official Letter No. 2/2021/CONEP/SECNS/MS for research with a virtual environment stage. Participants’ consent was obtained through an explanation of the objectives, reading, and signing of the Informed Consent Form. The following letters were used to ensure anonymity: W for woman, N for nurse, NT for nursing technician, MR for medical resident, and AP for attending physician, along with cardinal numbers according to the order of the interview.

### Theoretical-Methodological Framework

The theoretical-methodological framework used was the Grounded Theory (GT) approach, specifically the Straussian variant^(9)^.

### Type of Study

This qualitative study utilized the Consolidated Criteria for Reporting Qualitative Research (COREQ) as a guide to direct the research process report^(10)^.

### Study Setting

The study was conducted in the obstetric center of a teaching hospital in the western region of Paraná, a reference for childbirth for the 10th Regional Health Department of the state.

### Methodological Procedures

Participants were contacted either in person or via text message through the WhatsApp^®^ application. Selection was based on convenience, and all agreed to participate. Inclusion criteria were: a) first sample group (SG): women who had been assisted during vaginal birth by a multiprofessional team at the teaching hospital in western Paraná, between the first and second month postpartum, and were over 18 years old; b) second SG: nurses or nursing technicians working in the obstetric center of the study site with more than three months of experience; c) third SG: medical residents in obstetric training at the obstetric center in question for at least three months; d) fourth SG: attending obstetricians at the service.

### Data Collection and Organization

Data collection occurred between December 2019 and October 2020 through individual interviews conducted by a nursing student, guided by a researcher with expertise in the adopted framework, using a semi-structured script. The women and seven nursing professionals were interviewed in person, while the remaining participants were interviewed via the WhatsApp^®^ application. The interviews were conducted with a guarantee of privacy, preceded by a pilot test, audio-recorded, and began with the guiding question, “Talk about your perspective on the care provided to women during labor and childbirth.” The interviews had an average duration of 50 minutes.

Following this, other questions were used to guide the interviews. In the women’s SG, they discussed the care received from admission, the physical structure of the service, pain relief strategies, interventions received, the presence of a companion, and the place of birth, among other topics. In the nursing professionals’ SG, they explored the care provided to women, women’s knowledge about childbirth and delivery, confirmation of hypotheses about the topics discussed with them, appropriate versus inappropriate practices, and dialogue with the team for changes. In the medical residents’ and attending physicians’ SG, they reflected on medical practices, examining those misused, without scientific evidence, harmful or ineffective, and confirmed the hypotheses of the previous groups; additionally, they discussed professional training, the existence of care protocols, and openness to good practices.

The transcribed interview content was returned to the participants for comments and/or corrections, but none provided feedback. The study included 38 participants divided into four sample groups.

### Data Analysis

Data collection and analysis occurred simultaneously, in accordance with the stages of open, axial, and selective coding. In open coding, the data were analyzed line by line to establish initial categories. In axial coding, the categories were related to subcategories. Finally, in selective coding, the central category of the research was identified^(11)^.

In each stage of coding, the codes were grouped, regrouped, and ordered into categories and subcategories in a systematic process necessary for understanding the data and identifying the phenomenon studied. Diagrams and memos were constructed and supported the analysis, built from a paradigmatic perspective that describes the phenomenon through five elements: context, causal condition, intervening condition, strategy, and consequence. Data comparison, hypothesis confirmation, and theoretical explanation of the investigated phenomenon were achieved in the four SGs. Thus, theoretical saturation marked the conclusion of the data collection phase^(11)^.

## RESULTS

The central category, “Childbirth care by health professionals: conflicting practices in obstetrics”, is composed of six subcategories, presented according to the paradigmatic model.

### Limited Physical Structure for Adequate Care

The physical structure showed limitations in space and devices for care, such as having only one bathroom with a shower, which hinders adequate assistance to women during the parturition process. The service dealt with overcrowding, and privacy was not guaranteed.

[...] *very crowded, with stretchers in the hallway and women giving* [...] *birth.* (W7)

[...] *unable to provide adequate care due to the lack of structure,* [...] *there isn’t even a room available,* [...] *it should be much improved to* [...] *be more humanized* [...]. (MR5)

The service ensured the presence of a companion; however, due to the lack of space, a companion of one parturient compromised the privacy of another. The space limitation restricted the provision of adequate care and was used as a medical justification to transfer the woman from the room to the delivery room during the expulsive period.

[...] *when the sector is overcrowded,* [...] *the resident will perform an examination,* [...] *we ask the companion to leave* [...]. (NT13)

[...] *some attending physicians* [...] *say:* [...] *“I don’t allow the patient to give birth in the bed,* [...] *I don’t think it’s* [...] *humanized,* [...] *it’s cramped,* [...] *there’s a patient next to them,* [...] *the curtain is half open, and they will end up seeing,* [...] *hearing,* [...] *losing privacy”. They use this excuse.* (MR2)

While the companion’s presence compromised privacy on the one hand, it brought benefits on the other. Professionals sought to involve the companion in assisting the woman during labor by teaching pain relief strategies, such as performing massages and accompanying her during therapeutic baths, where the presence and words of encouragement provided emotional support.

[...] *guided massage for pain relief* [...] *assist when she goes for a therapeutic bath.* (E5)

### Observing Women’s (Lack of) Knowledge about Their Childbirth Process

The lack of knowledge about the childbirth process among the women served by the facility was noted in the participants’ statements. Fear of childbirth was linked to the lack of information and knowledge about the stages of labor, what to expect, and how the birth could happen. Pregnant women rarely visited the obstetric center to familiarize themselves with the place where they would experience childbirth. Therefore, the absence of information during prenatal care, combined with the unfamiliar environment, could explain requests for cesarean sections.

[...] *it took a long time,* [...] *the pain just increased,* [...] *I was asking for a cesarean section* [...] *I was terrified* [...]. (W4)

[...] *they don’t have much guidance about labor,* [...] *how it progresses, how long it takes* [...]. (MR4)

[...] *there is no scheduled visit* [...]. *It is very important for them* [...] *to be familiar with the environment.* (E3)

According to some professionals, having a birth plan indicates women’s knowledge about the childbirth process. On one hand, this tool was supported by medical residents and the nursing team. On the other hand, some attending physicians considered this plan an obstacle to interventions. In any case, its presentation was infrequent, probably due to the lack of knowledge among pregnant women about the birth plan and process.

[...] *labor flows much better* [if the woman has a birth plan] [...] *you know the patient has informed herself,* [...] *she is willing to have a natural birth* [...]. *We* [residents] *think like the nursing team* [...]. (MR4)

[...] *the birth plan* [...] *sometimes gets in the way* [...] *when it is necessary to intervene* [...]. *The doctor has the duty to guide* [...] *what is best for her* [...]. *These birth plan issues* [...] *I disagree* [...]. (MP3)

### Guiding and Caring during the Parturient Process

Due to women having insufficient information about the parturition process, both nursing professionals and medical residents sought to guide them regarding the childbirth process, non pharmacological methods for pain relief, birth positions, benefits of vaginal birth, and the importance of diet and hydration.

Regarding non-pharmacological methods for pain relief during the parturient process, therapeutic baths, freedom of choice for birthing positions, vocalization during labor, ambulation, massage, music therapy, aromatherapy, and other methods were primarily used and encouraged to provide a positive birth experience.

[...] *they have little information about labor* [...]. *Guide them on the benefits of vaginal birth,* [...] *birth positions* [...]. (MR1)

[...] *the stages she will go through,* [...] *pain,* [...] *bath,* [...] *vertical positions* [...]. *You create this bond,* [...] *teach and guide* [...] *positive thinking* [...]. *I tell them to* [...] *eat* [...]. (N4)

*Therapeutic bath,* [...] *music,* [...] *squatting, walking,* [...] *lower back massage* [...]. (NT9)

[...] *walking,* [...] *exercises* [...] *on the ball,* [...] *massage,* [...] *essential oils,* [...] *therapeutic bath,* [...] *non-analgesic measures for pain relief* [...], *making labor better.* (MR4)

### Guiding Professional Training

Professional training has significant importance in medical conduct and influences the understanding of what is appropriate or not in obstetric care. Longer training periods were shown to be relevant in justifying inappropriate medical practices.

[...] *they came from another era,* [...] *episiotomy* [...] *was routine* [...]. *They monitor labor in ways we consider today to be obstetric violence,* [...] *performing vaginal exams hourly,* [...] *especially the attending physicians* [...] *who have been there for many years.* (MR5)

[...] *episiotomy was mandatory in my training* [...] *to preserve the pelvic floor,* [...] *reduce the risk of sphincter injury* [...]. (MP4)

The role of nurses, particularly those with obstetric specialization, promoted good practices in childbirth care. Their practices were visible to the team due to their way of working and sharing their knowledge, which also contributed to the training of medical residents.

[...] *they guide exercises to help with contractions,* [...] *monitor fetal heartbeats,* [...] *progress of dilation* [...]. (AP2)

[...] *obstetric nurses* [...] *provide psychological support,* [...] *non-pharmacological analgesia,* [...] *activities that can help with the progress* [...] *of labor* [...]. (AP4)

[...] *obstetric nurses* [...] *have been working* [...] *sharing information with the rest of the team* [...] *about the latest studies related to labor.* (MR5)

### Arguing with Attending Physicians about New Practices

Some nurses and medical residents attempted to engage in dialogue with attending physicians regarding inappropriate care practices, questioning them based on the literature. The dialogue facilitated changes with some attending physicians; with others, there was no openness even for discussion, indicating resistance. Attending physicians often led medical residents to perform practices that conflicted with current recommendations and differed from the trainee’s beliefs.

[...] [with] *some professionals* [...], *there is no conversation or agreement,* [...] *some* [...] *are more accepting* [...] *when we* [...] *try to participate in defining the practices,* [...] *they define many practices,* [...] *you can try to question, but depending on the professional, it doesn’t help* [...]. (N3)

[...] *they are changing a little* [...], *but some attending physicians are not* [...], *they simply tell you to do it even if it’s against what you believe* [...]. *With dialogue, things improve a bit* [...]. (MR4)

Some attending physicians argued that the recommendations for new practices were utopian and derived from research and practices in first-world countries, therefore not adapted to the current reality. They asserted that professionals should base their actions on hospital experiences rather than theory.

[...] *I have more than 40 years of experience, and another has more than 30* [...]. *I am more experienced and those who teach, often,* [...] *know the theory, but the experience at the hospital door is different.* (MP3)

### Revealing Conflicting Obstetric Care: Good Practices and Obstetric Violence

The care provided during childbirth by most members of the nursing team and medical residents contrasts with that of the attending physicians. The latter, in particular, demonstrated resistance to implementing good practices, applied inappropriate practices, and violated the woman’s right to choose or compelled medical residents to do so.

*I had to walk to the delivery room. It felt like the baby was going to fall on the floor* [...]. (W5)

*Within the obstetric center, during my shift, I am always very clear:* [...] *I don’t want births outside the delivery room.* (AP4)

[...] *it can save the child’s life and help the mother if you know how to do* [Kristeller] [...]. *Just use one hand,* [...] *press the patient’s abdomen a little* [...]. (MP3)

There was a discrepancy between the theory supported by national and international recommendations for good practices and the routine medical practice in the service. Training, experiences, beliefs, and other factors guided the practice. Although professionals needed to develop their work activities according to international recommendations based on scientific evidence, many continued to maintain outdated and interventionist practices. Adding to this discrepancy was the absence of care protocols to guide practice in the service.

[...] *it is a team with older people who have difficulty accepting change,* [...] *most of them are attending physicians,* [...] *because they learned differently, they end up repeating what they learned over many years* [...]. (MR5)

[...] *I am not aware of any protocol regarding labor care* [...]. (MR2)

Regarding decisions, those of attending physicians prevailed over the choices of women and the opinions of the nursing team and medical residents about good practices. The justification was linked to the attending physician’s civil responsibility. They argued that they knew what was best for the parturient.

*The responsibility is yours. You are not going to share responsibility with the resident* [...]. (AP3)

[...] *the doctor’s perception greatly defines conduct and posture* [...]. *The hospital places him responsible for that patient* [...]. *It depends on the professional whether* [...] *the nurse participates or not.* (E6)

The accounts showed a clear conflict between the different practices of professionals in the studied service. Particularly, resistance to dialogue or changes was observed, especially by the attending physicians.

## DISCUSSION

The study highlights poor physical structure and a lack of privacy during the parturient process. These conditions favor the relocation of the woman to the delivery room during the active phase or expulsive period^(6)^, contrary to the WHO recommendation, which emphasizes a woman’s right to freely choose the place of birth^(2)^.

Maternity services should provide rooms and beds for pre-labor, labor, and postpartum to offer women in labor a familiar and safe environment, unlike a surgical room. Each room should have an individual bathroom for each woman in labor^(12)^, but the studied service has shared rooms and a single bathroom for all parturients.

In shared rooms, where a companion is present, partitions between beds are necessary to ensure and preserve that all women experience the childbirth process privately^(2)^. However, a woman’s right to have a companion throughout the hospitalization is violated in situations where the right of another to privacy prevails^(2,12)^.

It is emphasized that the presence of a companion has a positive impact on the childbirth experience. It provides emotional support, comfort, and tranquility for the parturient and contributes to the provision of non-pharmacological pain relief measures, such as massages and assistance in therapeutic baths, among other actions^(13)^.

It is noted that women receive scant guidance on normal childbirth, its stages, and benefits^(14)^. In this sense, it is inferred that a woman’s choice or request for a cesarean section may stem from fear of vaginal birth pain, equally reflecting the absence of adequate guidance about labor and childbirth^(15)^.

A visit to the maternity service (obstetric center) is recommended so that the pregnant woman can familiarize herself with the environment where the birth will take place, aiming to reduce anxiety and establish a trusting relationship with the service professionals^(16)^. However, such visits are almost nonexistent^(6)^.

The birth plan, a written document with detailed information about the woman’s choices presented to health professionals, promotes better communication between these professionals and the patient, potentially ensuring her autonomy^(17)^. This strategy should be encouraged throughout pregnancy^(2)^. However, its creation and submission are infrequent^(17,18)^, as women generally have insufficient education about childbirth^(15)^.

Even so, when a woman has knowledge about childbirth and presents a birth plan, there are still professionals who do not agree with or respect her autonomy regarding decisions about her own body^(19)^.

Regarding cesarean sections, Brazil has a high rate of this procedure, accounting for approximately 85% of births in private health services. In Paraná, the average cesarean rate across all services is 62%^(20)^, contrary to the WHO recommendation of 10% to 15%^(21)^. An additional factor contributing to the occurrence of cesarean sections is the legal support (Law 20127 of 2020), which guarantees women the right to choose the mode of delivery^(22)^.

However, this choice goes against the recommendations of official bodies, which emphasize the need to guide women during prenatal care about the indications for cesarean sections; they also highlight the necessity for professionals to persist and advise their performance only in cases of evident need, such as the impossibility of a vaginal birth confirmed by medical issues^(2,12)^.

Due to insufficient guidance during prenatal care, childbirth assistance professionals, particularly those in nursing, guide and assist women in labor according to current recommendations^(2,7,12,23)^. In the study, this included medical residents. The care provided by these professionals frequently involved the use of non-pharmacological methods for pain relief, such as lumbosacral massage, therapeutic baths, encouragement of ambulation, emotional support for the woman, free intake of fluids and food, and music therapy^(2,7,12)^.

However, there is still much room for improvement. For example, a study conducted in Ethiopia found that approximately 92% of women who had a vaginal birth experienced some form of disrespect during childbirth^(24)^. Some interventions during childbirth, currently considered inadequate or harmful, are still practiced, such as episiotomy, successive vaginal exams, the Kristeller maneuver, transferring the parturient to the delivery room, and the lithotomy position for birth^(2,7)^. In contrast, childbirth assistance provided by female professionals is considered a preventive factor against obstetric violence compared to assistance provided by male professionals^(24)^.

The role of obstetric nurses in the labor and delivery scenario has been highlighted for the implementation of recommended practices for vaginal childbirth supported by scientific evidence. This role, in addition to favoring women’s autonomy, promotes in-service education^(25)^. However, it is important to highlight the existence of critical factors that influence the availability of obstetric nurses in the Brazilian healthcare system, particularly in hospitals^(26)^.

The reports confirmed the resistance from the medical category to implementing humanized practices during labor and childbirth^(6)^, in contrast with the practices of nursing^(6,23,25)^ and medical residents. The attitudes of some professionals demonstrate the lack of incorporation of good practices, revealing weaknesses that compromise care for the parturient^(22)^ and contradicting international recommendations for a positive childbirth experience^(2)^.

In general, doctors disregard good practices and define their interventions as necessary to achieve favorable biological outcomes. Commonly, the pressure on obstetricians not to make mistakes is the justification they give for choosing these interventions^(27)^. However, this argument cannot override the recommendations of official bodies regarding appropriate and inappropriate practices.

As for inappropriate practices, the doctor’s imposition of the place and position for childbirth represents profound disrespect for the physiology of childbirth and the woman^(19,27)^. Therefore, transporting the parturient to a specific delivery room is considered untimely and unnecessary when labor is progressing without complications^(2)^. Instead of disadvantaging the parturient with this attitude, the team should offer her support regarding the choice of where and how to give birth, reinforcing the humanization of care^(12)^.

An example of imposition is found in a study where it was identified that, although women had refused an episiotomy, it was performed by a professional^(19)^. Another study reports that episiotomy and the lithotomy position were practiced in 100% of observed births^(27)^. Such practices disregard informed consent and violate the woman’s autonomy during childbirth^(2,19)^. Moreover, it is worth emphasizing that the routine and frequent use of episiotomy is contested by reference bodies for childbirth care, especially in women experiencing spontaneous vaginal birth^(2,12)^.

Concerning the Kristeller maneuver, it has been proscribed and should not be used as a means to facilitate labor^(2,12)^, as it is an unnecessary strategy capable of causing physical and psychological harm to the woman^(28)^. However, a study identified that childbirth assistance professionals consider the use of the Kristeller technique valid, ignoring its official proscription. Although the women who received this maneuver found it aggressive, their refusal was limited^(29)^. Additionally, there was a high prevalence of this procedure in a tertiary institution, which should have the capacity to handle obstetric emergencies without using untimely practices that violate the bioethical principles of autonomy and non-maleficence^(30)^.

There are practices that, when employed routinely and/or imposingly, disregard women’s rights and are characterized as obstetric violence^(2,12,31,32)^. These include neglecting women’s wishes, professional conduct based on generalized treatment in childbirth care with movement restriction, childbirth in the lithotomy position, excessive interventions, amniotomy, episiotomy, Kristeller maneuver, and non-consented or partially informed interventions, among other practices not recommended by national and international bodies.

The current care model predominantly relies on a hygienist legacy to execute hospital routines and practices, considering the doctor as the leader of the parturition process. As a result, the assistance and individual practices of these professionals become obstacles to instituting humanization in childbirth and delivery^(26)^. Therefore, to humanize childbirth and delivery care, it is essential to discontinue the archaic obstetric care model^(1)^ and abandon the inadequate techniques still practiced^(33)^.

For example, the Kristeller maneuver, officially prohibited, is still performed in health services during childbirth, sometimes without informing the woman about the procedure. Some professionals use gentle terms, referring to the maneuver as help in childbirth, while others believe that the risk associated with the procedure is due to a lack of technical training^(29)^.

The prominence attributed to humanization in care by current recommendations contrasts with the predisposition toward routines and practices that do not follow these recommendations^(34)^. It is worth noting that authoritarianism and hierarchy among the acting professional classes are the main factors contributing to obstetric violence^(35)^, as they disregard women’s human and reproductive rights^(19)^, even though these are provided for and ensured by current health recommendations.

In this regard, continuing education in service is highlighted as a relevant tool for improving the knowledge of practicing professionals, which can favor the implementation of care recommendations by all members involved in the childbirth scenario^(25)^.

Finally, it is important to remember that the current obstetric care model calls for the participation of obstetric nurses in the parturient process, as they seek humanization and practices that reduce inappropriate conduct^(2,12)^. In this sense, the autonomous role of obstetric nurses and the decision-making in conjunction with other team professionals^(23)^ can promote respectful, appropriate, and positive childbirth care for women.

### Study limitations

The study was limited to the reality of obstetric care in the obstetric center of a specific hospital in western Paraná. However, this service is a reference for high-risk births in the 10th Regional Health Department of the state, which includes 25 municipalities.

### Contributions to the fields of nursing and health

The study provides support for adequate childbirth care by nursing and health professionals, based on the principles of good practices. It particularly highlights obstetric nurses who, through their work, promote and encourage the implementation of appropriate practices, the empowerment of women, and the active participation of companions in the parturient process, in addition to contributing to the training of future professionals in the field.

## FINAL CONSIDERATIONS

Several aspects facilitated and/or triggered opportunities for inadequate practices or obstetric violence in childbirth care. These include the poor physical structure of the service under study, the insufficient knowledge and guidance of women about the childbirth and birth process, outdated professional training, the lack of updates in obstetrics, and the existence of a law that guarantees women the right to choose a cesarean section.

The insufficient knowledge of women and the lack of guidance for them about the childbirth and birth process during pregnancy interfere with their right to autonomy and empowerment within this process, compromising the possibility of making appropriate choices and presenting a birth plan. From this perspective, insufficient knowledge about childbirth is related to the choice of cesarean sections by women.

The central category of this study describes childbirth care by health professionals with conflicting practices in obstetrics, a discrepancy between good practices and obstetric violence, and between theory and practice. While some professionals base their practices on scientific evidence, others rely on teachings and experiences from the time of their training.

In light of the above, it is recommended that health education during prenatal care be promoted to ensure that the parturient has an appropriate birth. Actions from Brazilian and regional health programs, especially the application of guidelines from the Ministry of Health and WHO recommendations on childbirth and birth, should be implemented to guarantee good practices in the parturient process. Among these is the provision of care for usual-risk childbirth by obstetric nurses.
