# Supplementary material for: Childbirth care by health professionals: conflicting practices in obstetrics
Source: Rev Bras Enferm. 2024 Dec 16;77(6):e20230129. doi: 10.1590/0034-7167-2023-0129 (PMC11654509; doi:10.1590/0034-7167-2023-0129)
Supplement: Translation

# Translation: Assistência ao parto por profissionais de saúde: atuações conflitantes em obstetrícia

**DOI:** 10.1590/0034-7167-2023-0129pt

**Authors:** Greici Naiara Mattei, Gabriela Marcellino de Melo Lanzoni, Giovana Dorneles Callegaro Higashi, Taís Regina Schapko, Maria Aparecida Baggio

**Affiliations:** I Universidade Estadual do Oeste do Paraná. Cascavel, Paraná, Brasil; II Universidade Federal de Santa Catarina. Florianópolis, Santa Catarina, Brasil; III Universidade Federal de Santa Maria. Palmeira das Missões, Rio Grande do Sul, Brasil; IV Universidade Estadual do Oeste do Paraná. Foz do Iguaçu, Paraná, Brasil

**Keywords:** Assistência ao Parto, Enfermagem, Parturiente, Profissionais de Saúde, Obstetrícia.

## Abstract

**Objetivos:**

compreender a percepção de mulheres e profissionais de saúde sobre a assistência ao parto em um hospital-escola do oeste do estado do Paraná.

**Métodos:**

pesquisa qualitativa do tipo Teoria Fundamentada nos Dados, realizada em serviço de assistência obstétrica com 38 participantes (entre mulheres e profissionais de saúde) mediante entrevistas semiestruturadas.

**Resultados:**

as limitações da estrutura física e gestão da assistência, o incipiente conhecimento das mulheres sobre parto e o direcionamento e tomada de decisão de profissionais revelam práticas obstétricas conflitantes - um descompasso entre boas práticas e violência obstétrica. A formação em enfermagem obstétrica e participação ativa no cuidado associada à presença do acompanhante foram condições intervenientes e estratégias no processo.

**Considerações Finais:**

a assistência ao parto é caracterizada por atuações dicotômicas. Enquanto alguns profissionais sustentam suas práticas com base em evidências científicas, outros as pautam em ensinos e experiências relativas à época de sua formação.

## INTRODUÇÃO

O parto, um evento natural e fisiológico, foi influenciado pelo modelo biomédico e tornou se suscetível a diferentes práticas intervencionistas^(1)^. Em vista disso, para uma experiência de parto positiva, a Organização Mundial da Saúde (OMS) elaborou o Manual de Cuidados Intraparto, que contempla uma série de recomendações de práticas adequadas para os profissionais dos serviços obstétricos e para as mulheres em trabalho de parto^(2)^.

Práticas adequadas colocam a mulher como personagem principal do cuidado, reconhecem e consideram as suas necessidades e não são afetadas por imposições de profissionais de saúde ou da instituição na qual ela está inserida^(3)^. O cuidado durante o parto, quando centrado na mulher, permite-lhe decidir sobre a assistência obstétrica de acordo com as recomendações vigentes, proporcionando satisfação com o atendimento e uma experiência positiva do parto. Essas condições influenciam sua decisão de procurar novamente ou indicar o serviço de saúde obstétrico a outras gestantes^(4)^.

A ambiência do cenário de parto, a garantia da privacidade, o respeito às escolhas, a oferta de informações às mulheres para que possam fazer suas escolhas por meio do consentimento informado, a atuação dos profissionais de saúde envolvidos no processo de parturição, entre outros fatores, interferem na experiência do parto^(5)^. Atrelado a isso, a cooperação da equipe multiprofissional e a participação de profissionais atualizados e abertos à inovação têm contribuído para um novo modelo assistencial, orientado por práticas adequadas^(6)^.

Importa destacar que, no Brasil, embora se identifique a implementação das recomendações da OMS em algumas realidades, em outras persiste o modelo assistencial obstétrico não baseado em evidências científicas, que reflete uma assistência desrespeitosa. Em outras palavras, é notada a coexistência de práticas recomendadas e não recomendadas, sem embasamento científico, como a manobra de Kristeller, a episiotomia de rotina e a restrição de movimentos ou de alimentação^(7,8)^. Diante do exposto, questiona-se: Como mulheres e profissionais de enfermagem e de medicina compreendem a assistência ao parto em um hospital-escola do oeste do estado do Paraná?

## OBJETIVOS

Compreender a percepção de mulheres e profissionais de saúde sobre a assistência ao parto em um hospital-escola do oeste do estado do Paraná.

## MÉTODOS

### Aspectos éticos

Este estudo deriva de um macroprojeto intitulado “Rede Mãe Paranaense na perspectiva da usuária: o cuidado da mulher no pré-natal, parto, puerpério e da criança”, aprovado pelo Comitê de Ética em Pesquisa envolvendo Seres Humanos de uma instituição pública de ensino superior do norte do Paraná. Foi realizado de acordo com a Resolução n.º 466/2012 do Conselho Nacional de Saúde e Ofício n.º 2/2021/CONEP/SECNS/MS para pesquisas com etapa em ambiente virtual. A anuência dos participantes do estudo ocorreu mediante explicação dos objetivos, leitura e assinatura do Termo de Consentimento Livre e Esclarecido. Para garantir o anonimato, foram usadas as letras M para mulher, E para enfermeiro, TE para técnico de enfermagem, MR para médico residente e MP para médico plantonista, juntamente com número cardinal conforme ordem de entrevista.

### Referencial teórico-metodológico

A Teoria Fundamentada nos Dados (TFD), vertente Straussiana^(9)^, foi o referencial teórico metodológico.

### Tipo de estudo

Estudo qualitativo com utilização do *Consolidated Criteria for Reporting Qualitative Research* (COREQ) como guia para orientar o relatório do processo da pesquisa^(10)^.

### Cenário do estudo

Centro obstétrico de um hospital-escola da região oeste do Paraná, referência ao parto para a 10ª Regional de Saúde do Estado.

### Procedimentos metodológicos

Os participantes foram contatados presencialmente ou por mensagem de texto via aplicativo *WhatsApp^®^*. A seleção se deu por conveniência, e todos aceitaram participar. Foram critérios de inclusão: a) primeiro grupo amostral (GA): ter sido a mulher assistida durante o trabalho de parto vaginal por equipe multiprofissional de um hospital-escola do oeste do Paraná, estar entre o primeiro e segundo mês pós-parto e ter mais de 18 anos; b) segundo GA: ser enfermeiro ou técnico de enfermagem atuante no centro obstétrico do local do estudo e ter mais que três meses de experiência no serviço; c) terceiro GA: ser médico em formação obstétrica (residência) no centro obstétrico em questão por, no mínimo, três meses; d) quarto GA: ser médico obstetra plantonista do serviço.

### Coleta e organização dos dados

A coleta de dados ocorreu entre dezembro de 2019 e outubro de 2020 mediante entrevistas individuais realizadas por uma acadêmica de Enfermagem, orientada por uma pesquisadora com expertise no referencial adotado, utilizando um roteiro semiestruturado.

As mulheres e sete profissionais de enfermagem foram entrevistados presencialmente; e os demais participantes, por meio do aplicativo *WhatsApp^®^.* As entrevistas foram realizadas com garantia de privacidade, antecedidas por teste-piloto, audiogravadas e iniciadas com questão a norteadora “Fale sobre sua perspectiva acerca da assistência à mulher em trabalho de parto e parto”. Tiveram duração média de 50 minutos.

Após esta, outras questões foram utilizadas para conduzir as entrevistas. No GA das mulheres, elas versaram sobre a assistência recebida desde a admissão, estrutura física do serviço, estratégias de alívio da dor, intervenções recebidas, presença do acompanhante, local de parto, entre outros. No GA dos profissionais de enfermagem, exploraram a assistência realizada às mulheres, conhecimento das mulheres sobre parto e nascimento, confirmação de hipóteses sobre os temas abordados com elas, práticas adequadas versus inadequadas e diálogo com a equipe para mudanças. No GA dos médicos residentes e plantonistas, eles ponderaram sobre as práticas médicas, examinando as utilizadas de modo inadequado, sem evidências científicas, prejudiciais ou ineficazes e confirmaram hipóteses dos grupos anteriores; além disso, trataram da formação profissional, existência de protocolos de assistência e abertura para boas práticas.

O conteúdo da transcrição das entrevistas foi devolvido aos participantes para comentários e/ou correções, mas nenhum retornou. O estudo contou com 38 participantes, divididos em quatro grupo amostrais.

### Análise dos dados

A coleta e análise dos dados aconteceram simultaneamente, em conformidade com as etapas de codificação aberta, axial e seletiva. Na codificação aberta, os dados foram analisados linha por linha para estabelecer as categorias iniciais. Já na codificação axial, as categorias foram relacionadas às subcategorias. Por fim, na codificação seletiva, identificou-se a categoria central da pesquisa^(11)^.

Em cada etapa da codificação, os códigos foram agrupados, reagrupados e ordenados em categorias e subcategorias, num processo sistemático, necessário para a compreensão dos dados e identificação do fenômeno estudado. Diagramas e memorandos foram construídos e subsidiaram a análise, construída sob a perspectiva paradigmática, que descreve o fenômeno por meio de cinco elementos: contexto, condição causal, condição interveniente, estratégia e consequência. A comparação de dados, a confirmação de hipóteses levantadas e a explicação teórica acerca do fenômeno investigado foram alcançadas nos quatro GAs. Assim, a saturação teórica marcou o encerramento da etapa de coleta de dados^(11)^.

## RESULTADOS

A categoria central, “Assistência ao parto por profissionais de saúde: atuações conflitantes em obstetrícia”, é formada por seis subcategorias, apresentadas conforme modelo paradigmático.

### Tendo estrutura física limitada para um cuidado adequado

A estrutura física apresentava limitações de espaço e de dispositivos para assistência, como ter somente um banheiro com chuveiro, o que dificulta a assistência adequada à mulher no processo de parturição. O serviço mostrou lidar com superlotação, e a privacidade não era garantida.

[...] *muito cheio, cheio de maca no corredor e mulher ganhando* [...] *nenê.* (M7)

[...] *não consegue prestar uma assistência adequada pela falta de estrutura,* [...] *não tem nem um quarto disponível,* [...] *deveria melhorar muito para* [...] *ser mais humanizado* [...]. (MR5)

O serviço garantia a presença do acompanhante, porém, em razão da falta de espaço, o acompanhante de uma parturiente comprometia a privacidade de outra. O limite de espaço restringia a oferta de assistência adequada e era utilizado como justificativa médica para transferir a mulher do quarto para a sala de parto, no período expulsivo.

[...] *quando o setor está superlotado,* [...] *a residente vai fazer um toque,* [...] *a gente pede para o acompanhante se retirar* [...]. (TE13)

[...] *alguns médicos plantonistas* [...] *falam:* [...] *“eu não permito que a paciente tenha o parto no leito, [...] não acho [...] humanizado,* [...] *é apertado,* [...] *tem uma paciente do lado,* [...] *a cortina fica meio aberta, e vai acabar vendo,* [...] *ouvindo,* [...] *perde a privacidade”. Eles usam essa desculpa.* (MR2)

Se por um lado a presença do acompanhante comprometia a privacidade, por outro acarretava benefícios. Os profissionais buscavam envolver o acompanhante na assistência à mulher em trabalho de parto ensinando estratégias de alívio da dor, como realização de massagem e acompanhamento durante o banho terapêutico, em que a presença e palavras de incentivo configuravam apoio emocional.

[...] *orientava massagem para alívio da dor* [...] *auxiliar na hora que ela vai para o banho terapêutico.* (E5)

### Observando (des)conhecimento das mulheres acerca do seu processo de parto e nascimento

O desconhecimento sobre o processo de parto e nascimento pelas mulheres atendidas no serviço foi registrado nas falas dos participantes. O medo do parto foi atrelado à falta de informação e de conhecimento pela mulher sobre as fases do trabalho de parto, do que esperar e de como o parto poderia acontecer. As gestantes raramente realizavam visita ao centro obstétrico para conhecer o local onde iriam vivenciar o parto. Logo, a ausência de informações ainda no pré-natal, atrelada ao ambiente desconhecido, pode justificar pedidos por cesariana.

[...] *foi muito demorado,* [...] *as dores só aumentaram,* [...] *estava pedindo para fazer cesárea* [...] *senti muito medo* [...]. (M4)

[...] *não têm muita orientação sobre o trabalho de parto,* [...] *como* [...] *evolui, quanto tempo demora* [...]. (MR4)

[...] *não tem nenhuma visita programada* [...]*. É muito importante elas* [...] *terem conhecimento do ambiente.* (E3)

Conforme alguns profissionais, ter um plano de parto sinaliza conhecimento das mulheres sobre o processo de parto. Por um lado, essa ferramenta era apoiada por médicos residentes e pela equipe de enfermagem. Por outro lado, esse plano era considerado por alguns médicos plantonistas um obstáculo para intervenções. De toda forma, sua apresentação era pouco frequente, provavelmente devido ao desconhecimento das gestantes sobre o plano e processo de parto.

[...] *o trabalho de parto flui bem melhor* [se a mulher tem plano de parto] [...] *sabe que a paciente se informou,* [...] *está disposta a ter um parto normal* [...]*. A gente* [residentes] *pensa como a enfermagem* [...]. (MR4)

[...] *o plano de parto* [...] *às vezes atrapalha* [...] *quando é necessário intervir* [...]*. O médico tem a obrigação de orientar* [...] *para ela o que é melhor* [...]. *Essas questões de plano de parto* [...] *eu não concordo* [...]. (MP3)

### Orientando e cuidando durante o processo parturitivo

Em razão de as mulheres terem informações insuficientes sobre o processo de parturição, profissionais de enfermagem e médicos residentes buscavam orientá-las a respeito do processo de parto, métodos não farmacológicos para alívio da dor, posições para o nascimento, benefícios do parto normal e importância da dieta e ingesta hídrica.

Acerca dos métodos não farmacológicos para alívio da dor durante o processo parturitivo, eram principalmente utilizados e incentivados o banho terapêutico, liberdade de escolha de posição pela parturiente, vocalização no trabalho de parto, deambulação, massagem, musicoterapia, aromaterapia, entre outros, para proporcionar uma experiencia positiva de parto.

[...] *elas têm pouca informação sobre o trabalho de parto* [...]*. Orientar benefícios do parto normal,* [...] *posições do nascimento* [...]. (MR1)

[...] *fases pelas quais ela vai passar,* [...] *dor,* [...] *banho,* [...] *posições verticais* [...]*. Você consegue esse vínculo,* […] *ensina* [...] *e orienta* [...] *pensamento positivo* [...]*. Falo para* [...] *comer* [...]. (E4)

*Banho terapêutico,* [...] *música,* [...] *agachamento, caminhar,* [...] *massagem na região lombar* [...]. (TE9)

[...] *caminhada,* [...] *exercícios* [...] *na bola,* [...] *massagem,* [...] *óleos essenciais,* [...] *banho terapêutico,* [...] *medidas não analgésicas para alívio da dor* [...], *tornar o trabalho de parto melhor.* (MR4)

### Pautando a formação profissional

A formação profissional tem significativa importância na conduta médica e influencia o entendimento do que é adequado fazer, ou não, na assistência obstétrica. O maior tempo de formação mostrou-se relevante para justificar práticas médicas inadequadas.

[...] *vieram de outra época,* [...] *episiotomia* [...] *era rotina* [...]*. Acompanham o trabalho de parto como a gente considera hoje violência obstétrica,* [...] *fazendo o toque vaginal de hora em hora,* [...] *principalmente os plantonistas* [...] *que estão lá há muitos anos.* (MR5)

[...] *a episiotomia era obrigatória na minha formação* [...] *para preservar o assoalho pélvico,* [...] *diminuir os riscos de lesão de esfíncter* [...]. (MP4)

A atuação de enfermeiras, particularmente com especialização obstétrica, promoveu boas práticas na assistência ao parto. Suas práticas tinham visibilidade por parte da equipe, em razão de seu modo de atuar e de transmitir seus saberes, que também contribuíam com a formação de médicos residentes.

[...] *orientam os exercícios para ajudar nas contrações,* [...] *acompanham os batimentos cardíacos fetais,* [...] *evolução da dilatação* [...]. (MP2)

[...] *as enfermeiras obstétricas* [...] *dão o apoio psicológico,* [...] *analgesia não farmacológica,* [...] *atividades que possam ajudar na evolução [...] do trabalho de parto* [...]. (MP4)

[...] *as enfermeiras obstetras* [...] *tem atuado* [...] *passando as informações para o resto da equipe* [...] *sobre os estudos mais recentes em relação ao trabalho de parto.* (MR5)

### Argumentando com médicos plantonistas sobre novas práticas

Notou-se a tentativa de alguns enfermeiros e médicos residentes em dialogar com médicos plantonistas sobre práticas assistenciais inadequadas, questionando-os com base na literatura. Com alguns plantonistas, o diálogo permitia mudanças; com outros, não havia abertura nem para o diálogo, denotando resistência. Já os médicos residentes eram conduzidos por plantonistas a realizar práticas discordantes das recomendações vigentes e contrárias àquelas em que o profissional em formação acreditava.

[...] [com] *alguns profissionais* [...], *não tem conversa e acordo,* [...] *alguns* [...] *aceitam melhor* [...] *quando a gente* [...] *tenta participar da definição das condutas,* [...] *muitas condutas eles que definem,* [...] *pode até tentar questionar, mas, dependendo do profissional, não adianta* [...]. (E3)

[...] *eles estão mudando um pouco* [...]*, mas tem alguns plantonistas que não* [...]*, simplesmente mandam você fazer mesmo que seja contra o que você acredita* [...]*. Com diálogo, as coisas melhoram um pouco* [...]. (MR4)

Acerca das recomendações de novas práticas, alguns plantonistas argumentavam que eram utópicas e advindas de pesquisas e práticas de países de primeiro mundo, portanto não adaptadas à realidade em questão. Eles defendiam que os profissionais deveriam se pautar pelas vivências de porta de hospital e não pela teoria.

[...] *tenho mais de 40 anos de experiência, tem outro que tem mais de 30* [...]*. Sou mais antigo e o pessoal que dá aula, muitas vezes,* [...] *sabem a teoria, mas a vivência de porta de hospital é diferente.* (MP3)

### Revelando assistências obstétricas conflitantes: boas práticas e violência obstétrica

A atuação na assistência ao parto por parte da maioria dos membros da equipe de enfermagem e de médicos residentes contrasta com a atuação de médicos plantonistas. Estes últimos, principalmente, manifestavam resistência à implementação de boas práticas, aplicavam práticas inadequadas e violavam o direito de escolha da mulher ou impeliam médicos residentes a isso.

*Tive que ir andando para a sala de parto. Parecia que o bebê ia cair no chão* [...]. (M5)

*Dentro do centro obstétrico, no meu plantão, eu sempre sou muito claro:* [...] *não quero parto fora da sala de parto.* (MP4)

[...] *pode salvar a vida da criança e ajudar a mãe se você souber fazer* [Kristeller] [...]. *Coloca uma mão só,* [...] *pressiona um pouquinho a barriga da paciente* [...]. (MP3)

Havia descompasso entre a teoria sustentada pelas recomendações nacionais e internacionais de boas práticas e a prática médica rotineira no serviço. A atuação era orientada pela formação, experiências, vivências, crenças, entre outros fatores. Embora os profissionais necessitassem desenvolver suas atividades laborais de acordo com as recomendações internacionais baseadas em evidências cientificas, muitos acabavam mantendo a atuação de outrora e implementavam práticas desatualizadas e intervencionistas. Soma-se a esse descompasso a inexistência de protocolos assistenciais para orientar a prática assistencial no serviço.

*[...] é uma equipe com pessoas mais antigas e que têm dificuldade para aceitar a mudança, [...] a maioria são os plantonistas, [...] por eles terem aprendido de outra maneira, acabam repetindo o que aprenderam durante muitos anos [...].* (MR5)

*[..] Não tenho conhecimento de nenhum protocolo referente a assistência ao trabalho de parto [...].* (MR2)

Quanto às decisões, as de médicos plantonistas prevaleciam sobre as escolhas das mulheres e opiniões da equipe de enfermagem e dos médicos residentes acerca das boas práticas. A justificativa era atrelada à responsabilidade civil do plantonista. Eles argumentavam entender o que era melhor para a parturiente.

*Responsabilidade é tua. Você não vai dividir reponsabilidade com residente* [...]. (MP3)

[...] *a percepção dele* [médico] *define muito conduta e postura* [...]. *O hospital o coloca responsável por aquele paciente* [...]. *Depende do profissional a* [...] *participação ou não do enfermeiro.* (E6)

Os relatos mostraram haver claro conflito entre as diferentes atuações dos profissionais no serviço estudado. De modo particular, observou-se resistência ao diálogo ou a mudanças, particularmente por parte dos plantonistas.

## DISCUSSÃO

O estudo evidencia estrutura física precária e ausência de privacidade durante o processo parturitivo. Essas condições favorecem o deslocamento da mulher para sala de parto em fase ativa ou período expulsivo^(6)^, contrapondo recomendação da OMS, que destaca o direito da mulher à livre escolha sobre o local do parto^(2)^.

Os serviços de parto devem dispor de quartos e camas pré-parto, parto e puerpério para conferir à mulher em trabalho de parto um ambiente familiar e seguro, dessemelhante a uma sala cirúrgica. O quarto deve ter banheiro individual para cada mulher em trabalho de parto^(12)^, mas o serviço estudado dispõe de quartos compartilhados e banheiro único para todas as parturientes.

Nos quartos compartilhados, onde o acompanhante está presente, é preciso garantir e preservar que todas as mulheres vivenciem o processo de parto de forma privativa, utilizando divisórias entre os leitos^(2)^. Contudo, o direito de uma mulher de estar acompanhada durante todo o internamento é descumprido em situações em que prevalece o direito da outra à privacidade^(2,12)^.

Salienta-se que a presença do acompanhante tem aspecto positivo na experiência do parto, permitindo a oferta de apoio emocional, conforto e tranquilidade para a parturiente, além de contribuir com provimento de medidas não farmacológicas para alívio da dor, como massagens, auxílio no banho terapêutico, entre outras ações^(13)^.

Constata-se que as mulheres tem orientações exíguas sobre o parto normal, suas etapas e benefícios^(14)^. Nesse sentido, infere-se que a escolha ou pedido da mulher pela cesariana pode advir do medo da dor do parto vaginal, sendo igualmente reflexo da ausência de orientações adequadas acerca do trabalho de parto e parto^(15)^.

A visita ao serviço de parto (centro obstétrico) é recomendada para que a gestante conheça previamente o ambiente onde será realizado o parto, com o intuito de abreviar a ansiedade e estabelecer uma relação de confiança com os profissionais do serviço^(16)^. Entretanto, essas visitas são quase inexistentes^(6)^.

O plano de parto, apresentado aos profissionais de saúde por meio de documento escrito com informações detalhadas das escolhas da mulher, promove melhor comunicação entre esses profissionais e a mulher, podendo garantir-lhe autonomia^(17)^. Trata-se de uma estratégia que deve ser encorajada ao longo da gestação^(2)^. No entanto, sua elaboração e entrega são pouco frequentes^(17,18)^, porque, em geral, as mulheres têm instrução insuficiente sobre parto^(15)^.

Mesmo assim, quando a mulher dispõe de conhecimentos sobre o parto e apresenta um plano de parto, há ainda profissionais que não concordam ou não respeitam a autonomia dela no que concerne às decisões sobre seu próprio corpo^(19)^.

No tocante à cesariana, o Brasil possui alto índice desse procedimento, correspondendo a aproximadamente 85% dos partos nos serviços privados de saúde. Já no Paraná, a média de cesáreas em todos os serviços é de 62%^(20)^, contrariando o que recomenda a OMS, entre 10% e 15%^(21)^. Fator adicional para a ocorrência da cesariana é o respaldo jurídico (Lei 20127 de 2020), que garante à mulher a escolha da via de parto^(22)^.

Todavia, essa escolha vai na contramão das recomendações de órgãos oficiais, que advertem sobre a necessidade de orientar a mulher no pré-natal quanto à indicação de cesariana; além disso, destacam a imprescindibilidade de os profissionais persistirem e orientarem sua realização somente em casos de necessidade evidente, como na impossibilidade de um parto vaginal, confirmada por questões médicas^(2,12)^.

Por causa da incipiente orientação no pré-natal, os profissionais de assistência ao parto, particularmente os da enfermagem, orientam e assistem as mulheres em trabalho de parto de acordo com as recomendações em voga^(2,7,12,23)^. No estudo, isso incluiu os médicos residentes. A assistência realizada por esses profissionais retrata o uso frequente de métodos não farmacológicos para alívio da dor, como massagem lombossacral, banho terapêutico, incentivo a deambulação, apoio emocional à mulher, livre ingestão de líquidos e alimentos e musicoterapia^(2,7,12)^.

Entretanto, ainda há muita margem para avanço. Por exemplo, um estudo realizado na Etiópia identificou que aproximadamente 92% das mulheres que tiveram parto vaginal sofreram algum tipo de desrespeito no momento parto^(24)^. Algumas intervenções no parto, atualmente consideradas inadequadas ou prejudiciais, ainda são praticadas, tais como episiotomia, toques vaginais sucessivos, manobra de Kristeller, transferência da parturiente para sala de parto e posição litotômica para parir^(2,7)^. Em contraposição, a assistência ao parto realizada por profissional de sexo feminino é considerada um fator de prevenção à violência obstétrica quando comparada à assistência ofertada por profissionais de sexo masculino^(24)^.

A atuação de enfermeiras obstétricas no cenário do trabalho de parto e parto tem recebido destaque para a implementação de práticas recomendadas de assistência ao parto normal, respaldadas por evidências científicas. Essa atuação, além de favorecer a autonomia das mulheres, promove educação em serviço^(25)^. Contudo, importa destacar a existência de fatores críticos que influenciam a disponibilidade de enfermeiras obstétricas no sistema de saúde brasileiro, particularmente nos hospitais^(26)^.

Os relatos confirmaram a resistência por parte da categoria médica à implementação de práticas humanizadas durante o trabalho de parto e parto^(6)^, em contraste com as práticas da enfermagem^(6,23,25)^ e de médicos residentes. As atitudes de alguns profissionais demonstram a não incorporação das boas práticas, revelando fragilidades que comprometem a assistência à parturiente^(22)^ e contrariando recomendações internacionais para uma experiência de parto positiva^(2)^.

Em geral, os médicos desconsideram as boas práticas e definem suas intervenções como necessárias para obtenção de resultados biológicos favoráveis. Comumente a pressão sobre os médicos obstetras de não errar é a justificativa que eles dão para escolher essas intervenções^(27)^. Todavia, esse argumento não pode sobrepor-se às recomendações dos órgãos oficiais acerca das práticas adequadas e inadequadas.

Quanto às práticas inadequadas, a imposição do médico determinando o local e posição de parir representa profundo desrespeito à fisiologia do parto e à mulher^(19,27)^. Por isso, o transporte da parturiente para uma sala de parto específica é considerado como intempestivo e dispensável quando o trabalho de parto está evoluindo sem intercorrências^(2)^. Em vez de desfavorecer a parturiente com essa atitude, cabe à equipe oferecer-lhe apoio quanto à escolha de onde e de como parir, reforçando a humanização da assistência^(12)^.

Um exemplo de imposição é encontrado em estudo no qual se identificou que, embora mulheres tivessem recusado a episiotomia, ela foi realizada pelo profissional^(19)^. Outra pesquisa afirma que foram praticadas a episiotomia e a posição litotômica em 100% dos partos observados^(27)^. Tais condutas desconsideram o consentimento informado e descumprem a autonomia da mulher diante da parturição^(2,19)^. Além disso, vale reforçar que o uso habitual e frequente da episiotomia é contestado por órgãos de referência à assistência ao parto, sobretudo em mulheres que vivenciam o parto vaginal espontâneo^(2,12)^.

Quanto à manobra de Kristeller, ela foi proscrita e não deve ser utilizada como meio facilitador do trabalho de parto^(2,12)^, visto que é uma estratégia dispensável, capaz de causar danos físicos e psicológicos à mulher^(28)^. Entretanto, estudo identificou que profissionais de assistência ao parto consideram válido o uso da técnica de Kristeller, ignorando sua proscrição oficial. Embora as mulheres que receberam tal manobra a tenham considerado agressiva, sua recusa foi limitada^(29)^. Além disso, constatou-se elevada prevalência desse procedimento em instituição terciária, que teria capacidade resolutiva para enfrentar emergências obstétricas sem o uso de práticas intempestivas que ferem os princípios bioéticos de autonomia e não maleficência^(30)^.

Há condutas que, empregadas de forma rotineira e/ou impositiva, desconsideram os direitos das mulheres e caracterizam-se como violência obstétrica^(2,12,31,32)^. Entre elas, destacam-se o descaso quanto aos desejos das mulheres, atuação profissional baseada em tratamento generalizado na assistência ao parto e com restrição de movimentos, parto em posição litotômica, excesso de intervenções, amniotomia, episiotomia, manobra de Kristeller, intervenção não consentida ou aceita com informações parciais, entre outras práticas não recomendadas por órgãos nacionais e internacionais.

O modelo assistencial atual apoia-se, majoritariamente, em um legado higienista para executar as rotinas e práticas hospitalares, considerando o médico como preceptor do processo de parturição, de modo que a assistência e a prática individual desses profissionais tornam-se obstáculo para se instituir a humanização no parto e nascimento^(26)^. Logo, para humanizar a assistência ao parto e nascimento, é fundamental descontinuar o modelo assistencial obstétrico arcaico^(1)^, abdicando das técnicas inadequadas ainda praticadas^(33)^.

Por exemplo, a manobra de Kristeller, oficialmente proibida, ainda é executada nos serviços de saúde durante o parto, em alguns casos sem informar a mulher sobre o procedimento. Alguns profissionais utilizam termos gentis, referindo-se à manobra como uma ajuda ao parto, enquanto outros acreditam que o risco relacionado ao procedimento é associado à falta de treinamento técnico^(29)^.

A notoriedade atribuída à humanização na assistência pelas recomendações atuais contrasta com a predisposição de rotinas e condutas que não seguem tais recomendações^(34)^. Vale destacar que o autoritarismo e a hierarquia entre as classes profissionais atuantes são os principais fatores para a violência obstétrica^(35)^, visto que desconsideram os direitos humanos e reprodutivos das mulheres^(19)^, embora previstos e assegurados pelas recomendações de saúde vigentes.

Sobre isso, a educação continuada em serviço é apontada como uma importante ferramenta para a melhoria dos conhecimentos dos profissionais da prática, o que pode favorecer a implementação das recomendações de assistência ao parto por todos os membros que atuam no cenário de parto^(25)^.

Por fim, vale lembrar que o modelo de assistência obstétrica atual solicita a participação de enfermeiras obstétricas no processo de parturição por buscarem humanização e práticas que reduzem as condutas inadequadas^(2,12)^. Nesse sentido, a atuação autônoma de enfermeiras obstétricas e a decisão das condutas em conjunto com os demais profissionais da equipe^(23)^ podem promover uma assistência ao parto respeitosa, adequada e positiva para a mulher.

### Limitações do estudo

O estudo limitou-se à realidade de assistência obstétrica de um centro obstétrico de determinado hospital do oeste do Paraná. Contudo, o serviço é referência para partos de alto risco da 10ª Regional de Saúde do Estado, que comporta 25 municípios.

### Contribuições para a área da enfermagem e saúde

O estudo oferece subsídios para uma assistência adequada ao parto por profissionais de enfermagem e de saúde, nos princípios das boas práticas. Sobretudo, confere destaque às enfermeiras obstétricas que, por meio da sua atuação, favorecem e estimulam a implementação de práticas adequadas, o empoderamento da mulher e a participação ativa dos acompanhantes no processo de parturição, além de contribuírem com a formação de futuros profissionais da área.

## CONSIDERAÇÕES FINAIS

Alguns aspectos facilitaram e/ou desencadearam oportunidade de práticas inadequadas ou violência obstétrica na assistência ao parto. Entre eles, estão a precária estrutura física do serviço em estudo, o incipiente conhecimento e orientação das mulheres sobre o processo de parto e nascimento, a formação profissional em moldes antigos, a ausência de atualização em obstetrícia e a existência de uma lei que garante à mulher o direito de escolha por cesárea.

O incipiente conhecimento das mulheres e a ausência de orientações para elas sobre o processo de parto e nascimento durante a gestação interferem tanto no direito à autonomia e no empoderamento dentro desse processo, comprometendo a possibilidade de escolhas adequadas, quanto na apresentação de um plano de parto. Dessa perspectiva, o insuficiente conhecimento acerca do parto se relaciona com a escolha de cesárea pelas mulheres.

A categoria central deste estudo descreve uma assistência ao parto por profissionais de saúde com atuações conflitantes em obstetrícia, num descompasso entre boas práticas e violência obstétrica, entre teoria e prática. Enquanto alguns profissionais sustentam suas práticas com base em evidências científicas, outros se pautam em ensinos e experiências relativos à época de sua formação.

Diante do exposto, indica-se a promoção de educação em saúde ainda no pré-natal para garantir que a parturiente tenha um parto adequado. Recomenda-se a implementação de ações de programas de saúde brasileiros e regionais, sobretudo a aplicação de diretrizes do Ministério da Saúde e recomendações da OMS acerca do parto e nascimento para garantia de boas práticas no processo de parturição. Dentre elas, encontra-se a oferta de assistência ao parto de risco habitual por enfermeiras obstétricas.
